# Benefits of managerial overconfidence for corporate digital transformation: Evidence from China

**DOI:** 10.1371/journal.pone.0314231

**Published:** 2024-11-21

**Authors:** Yi Jin, Chuxin Chen, Bo Liu

**Affiliations:** 1 Business School, Southwest Minzu University, Chengdu, China; 2 School of Economics and Management, Southwest University of Science and Technology, Mianyang, China; The university of Chenab, PAKISTAN

## Abstract

This article studies how managerial overconfidence shapes a firm’s digital transformation and unpacks the pathway and the boundary condition. Using a sample of Chinese listed firms between 2011 and 2022, we find that managerial overconfidence exhibits a positive relationship with corporate digital transformation and the relationship is mediated by R&D investment. In addition, we demonstrate that compared to state-owned companies, R&D investment has a more significant positive effect on digital transformation in non-state-owned companies. This study contributes novel insights about the consequences of cognitive biases of top managers and extends the studies for investigating top managers in the digital transformation age. The findings offer implications for top managers who have to fully recognize the impact of overconfidence on the development of the companies and the impact of overconfident executives on corporate digital transformation depending on R&D investment to create value. In addition, this study also reminds Chinese policymakers to improve the efficiency of converting R&D investment into digital transformation in state-owned enterprises.

## Introduction

Digital transformation is defined as an organizational transformation process precipitated by various digital technologies (e.g., artificial intelligence, the Internet of Things) [1, 2]. Drawing from convergence and generavity features of digital technologies, corporate digital transformation may improve resource allocation, disrupt the traditional value creation chain, and induce new quality productive forces [3–5]. Prior studies argue that such transformation asks for fundamental changes in business processes, organizational structure, and capabilities and then provides valuable understandings regarding how to transform under uncertainty (e.g., [6–8]). However, in recent years, management research and real-world businesses also found that most companies struggle to implement transformation effectively for multidimensional reasons [9], such as unclear digital strategic goals, lack of digital resources, and in-consistent transformation cognition [9, 10].

Despite the conflicts about digital transformation implementation pathways, there is a fairly strong consensus that digital transformation is a top management project [11, 12]. A successful business case for digital transformation is Schneider Electric. Due to the top management’s perception of digital technology integration and digital ecosystem, Schneider Electric positioned itself as an industry application expert at the beginning of its transformation, improving operational production efficiency, and thereby achieving transformation from 0 to 1 [13].

The classical decision-making theory holds that as rational decision-makers, top managers aim to maximize organizational benefits. However, behavioral decision-making theory suggests that people do not choose the optimal decision rationally, and the bounded rational behavior of executives in a dynamic environment is increasingly prominent [14]. Upper echelons theory further argues that corporate strategic decisions are often influenced by the characteristics of managers [15]. Previous studies reveal that the characteristics of executives such as gender, education, and compound functional backgrounds [16–18] exert impacts on corporate digital transformation strategic decision-making. Some studies have also found that executive teams can have an impact on corporate digital innovation capability, digital orientation, and effective digitization [11, 19, 20]. However, there is little literature on the significant impact of cognitive biases generated in self-evaluation on digital transformation.

Overconfidence is a cognitive and behavioral bias, which is viewed as an important departure from the *homo oeconomicus* paradigm [21]. To develop a specific picture of overconfidence, understanding its related but distinct concepts (i.e., confidence and underconfidence) is important. As suggested by [22], confidence is defined as the trust or faith in something (e.g., the own capabilities or knowledge), while underconfidence and overconfidence come from inaccuracies in one’s confidence judgment. Underconfidence underestimates one’s judgment, while overconfidence is about excessive estimation. Together, such biased beliefs will impact agents’ effort decisions but seemingly apply to different settings. Confidence and underconfidence are widely studied in individual decision-making research, such as salespersons, general leaders, and entrepreneurs. To some degree, confidence is often being used interchangeably with (or as a constituent component of) “self-efficacy” to depict the beliefs in their capability. However, the impacts of overconfidence are relevant for macroeconomics and are studied in a broad range of managerial decisions, such as acquisition, cashing holdings, and innovation. Given that there is a fairly strong consensus that people in high positions are prone to be overconfident and that digital transformation involves top managers’ efforts, we aim to explore the net effects of the overconfidence of top management teams on digital transformation.

To our knowledge, Zhu, et al. [23] links CEO overconfidence with positive corporate digital transformation, because overconfident CEOs exhibit more optimism than the average about the future which leads them willing to mobilize corporate resources to develop challenging digital transformation projects. They also found that digital finance has the potential to free businesses from financial restraints and thus amplify the effect of CEO overconfidence and spur digital transformation. Both the CEO and overconfidence studies significantly contribute to our understanding of digital transformation, but a notable gap persists in ignorance in the top management team, because despite the decisive influence of the CEO in modern organizational management, many decisions are made jointly by the top management team. This gap constrains our understanding of how the overconfidence of top managers impacts transformation implementation pathways and facilitates organizational digital transformation. An in-depth and holistic sense of top management teams’ overconfidence and their corresponding actions is essential to understand cognitive biases in collective decision-making processes [9].

To address the theoretical gap and extend the prior literature on digital transformation, this article posits that digital transformation will depend in part on top managers’ cognitive ability to correctly evaluate the degree and importance of the uncertainty they face. In particular, we emphasize one important type of cognitive bias, overconfidence will encourage top managers to undertake more pressure from risky organizational change, tend to place more resources on R&D activities, and thereby facilitate digital transformation. In addition, we argue that, compared to non-state-owned companies, state-owned companies have more resources and are more likely to invest in digitalization under institutional pressure. However, there is an efficiency paradox in state-owned enterprises, which leads to lower efficiency in converting R&D investment into digital outcomes. Thus, we predict that the positive effect of R&D investment on digital transformation is less positive for state-owned firms than for non-stated firms.

We test our predictions using the empirical setting of China’s public firms in 11 years from 2011 through 2021. Consistent with our expectations, we found that managerial overconfidence is positively associated with corporate digital transformation, and the underlying mechanism is mediated by R&D investment. Moreover, we found that the relationship between R&D investment and digital transformation is less positive in state-owned companies. Our findings are particularly convincing because the results are in line with various robustness checks using alternative measures of managerial overconfidence and considering time lags.

This article primarily contributes to digital transformation studies by examining whether managerial cognitive bias might inform corporate digital transformation and offering new insights for corporations seeking to foster digitalization. While previous digital transformation studies have examined some characteristics of top managers, this article is among the first to confirm the positive role of overconfidence of the top management team in this context, which is beyond Zhu, et al. [23] and reveals the impact of overconfidence in collective decision-making processes. Second, this article reveals the underlying mechanism in elucidating managerial overconfidence enabling digital transformation, going beyond the studies that focus on how overconfident managers influence organizational strategic decision-making [24]. Our finding shows that overconfident top managers overestimate their ability and performance, are inclined to invest in R&D activities and thereby advance the digital transformation of a company. Third, by focusing on state ownership, this article also complements existing studies (e.g., [25]) by showing that the impact of overconfidence in top management teams on digital transformation may vary in different ownership, which suggests efficiency problems in state-owned companies.

## Literature review and hypotheses development

In this section, we first introduce the research status quo on digital transformation and managerial overconfidence to explain why predicting digital transformation from the perspective of overconfidence is insightful. Then, based on theoretical deduction, we link managerial overconfidence and digital transformation to develop the main assumption and next, we introduce R&D investment and state ownership to further unpack the relationship between managerial overconfidence and digital transformation.

### Digital transformation

Digital transformation can overturn corporate innovation and operational models and has a wide-ranging impact on corporate survival and development. Previous studies have affirmed the positive effects of digital transformation on resource allocation efficiency, innovation, and sustainable performance [3, 7, 26]. Because digital transformation is a rewarding but risky pursuit [27], scholars gradually pay attention to the driving factors of digital transformation. Lyytinen and Rose [28] argue that the rapidly changing market environment has disrupted the existing equilibrium of market competition, and companies tend to engage in digital transformation to strengthen their connection with the market. Xu and Liu [29] believe that economic policy uncertainty promotes the digital transformation of enterprises through three paths: reduced operational efficiency, increased financing constraints, and increased systemic risks. Zhang, et al. [30] believe that supply chain finance promotes digital transformation through three paths: reducing information asymmetry, mitigating financial constraints, and improving total factor productivity.

External perspective research focuses on the factors from the external perspectives of the external environment and stakeholders. However, digital transformation is not only a technical issue but more importantly, a strategic choice [31]. Digital transformation, as an organizational change, is naturally more susceptible to the influence of internal executive teams. Previous upper echelons studies show that as the decision-making body of corporate strategy, the characteristics of the executive team will have a significant impact on the choice of corporate strategy and digitalization [14]. For example, Li and Shao [19] found the diversity and average education level of top management teams facilitate corporate digital orientation, while average age and tenure have a counterproductive effect. Zhu, et al. [32] demonstrate that the digital technology background of CEOs can better propel corporate digital transformation via digital learning and career experience.

Previous studies help us to understand how top managers drive digital transformation. However, academic attention still needs to be paid to more characteristics of top managers. In particular, overconfidence, which is seen as the mother of cognitive biases [33], manages the cognitive faith toward digital transformation, which may affect digital resource allocation and implementation [23]. Therefore, this article aims to explore whether overconfidence of the executive team will have an impact on digital transformation, and clarify the mechanism of the relationship between the two.

### Managerial overconfidence

Overconfidence is defined as a psychological trait that an individual inflates estimates regarding the accuracy of one’s beliefs, knowledge, abilities, levels of control, and performance [34, 35], which will affect how individuals view and respond to the situations they face. Psychologists have long related overconfidence with the pejorative term “hubris” in reference to the upward bias in the assessment of their abilities, and thus some scholars employ the two terms interchangeably [35]. While findings from social psychology indicate that overconfidence may be not a stable trait and may develop over time through biased self-attribution and power structures [34, 36], management scholars study overconfidence in the context of entrepreneurs and executives (e.g., CEO), to explain strategic decision-making, investment, innovation, performance, and value of a company.

Although research conclusions are inconsistent, scholars focus on the better-than-average effect and miscalibration effect when making theoretical reasoning [37]. Better-than-average effect comes from overconfident people’s belief that they can act better than average in a wide variety of tasks [38]. The miscalibration effect comes from overconfident people’s optimistic prediction toward the variance with respect to uncertain events [37]. Therefore, such effects support them to bear more pressure to conduct challenging and uncertain tasks and engage in aggressive investment activities.

Based on the two effects, only a few scholars have paid attention to the impact of executive overconfidence on corporate digitalization in the context of the digital economy. Yang, et al. [39] found that managerial overconfidence weakens the impact of digital transformation on corporate innovation because overconfident managers are prone to making expansionary investments, increasing the probability of the company falling into financial difficulties, which is not conducive to innovation. Zhu, Li, and Ma [23] propose a positive relationship between CEO overconfidence and corporate digital transformation and incorporate digital finance into the conceptual model. Considering there is little literature on the impact of executive overconfidence on digital transformation, the research conclusions are inconsistent. Therefore, exploring the relationship between managerial overconfidence and corporate digital transformation and the internal mechanism is increasingly significant and valuable.

### Managerial overconfidence and digital transformation

We posit that managerial overconfidence has an impact on corporate digital transformation because of the following three reasons. First, overconfident managers exhibit a greater tolerance for failure, which leads to aggressive and risky decisions. Managerial overconfidence involves cognitive mechanisms of assigning probabilities to possible outcomes and assessing their relative attractiveness [40]. Overly positive cognitive perceptions of themselves and their situations lead confident managers to believe that certain outcomes are more feasible than they are. Therefore, overconfident managers inaccurately assess the risks they should bear and are inclined to undertake more [24, 41]. Digital transformation—which causes discontinuous organizational changes for a company—is risky and challenging. We therefore suppose managerial overconfidence to be potentially important for such undertakings. In the process of digital transformation, this strategic risk-taking is crucial, as digital transformation often comes with uncertainty and technological risks. Overconfident executives may be more proactive in driving innovation and change [42], thereby accelerating the firm digital transformation process [23].

Second, overconfident managers typically make aggressive investments. Previous research confirms that overconfident managers have a high propensity to utilize cash for strategic investment (e.g., acquisition) rather than cash holding [43]. Overconfident executives may be more willing to invest in new technologies and digital infrastructure because they have higher confidence in their judgment and predictive abilities. Such resource investment helps businesses quickly adopt and integrate advanced digital technologies, thereby driving digital transformation.

Third, overconfident executives exercise influence attempts on followers. Confident managers are regarded as competent and capable by others [44]. Due to confidence in their judgments and decisions, resolute decision-making that overconfident managers make can help followers quickly sense transformation determination and understand the transformation goals of a company, reduce hesitation and procrastination, and thus accelerate the pace of digital transformation and decrease internal resistance. Moreover, due to overestimation, overconfident managers tend to express expectations of positive outcomes. Thus, overconfidence enables them well present an idealized image of the future and inspire followers to implement actions [44]. In this respect, effective vision and emotional contagion inspire employees to believe that digital transformation is feasible and worth investing in and motivate employees to overcome difficulties in change. Thus, we predict the following:

H1. *Managerial overconfidence is positively associated with the digital transformation of a company*.

### Mediating role of R&D investment

R&D investment, as a strategic source for corporate survival and development, sets a company apart from its competitors. Overconfidence, as an individual characteristic of top managers, affects their decision-making and subsequently affects the firm value creation actions. Overconfident managers are more adventurous and thus tend to pursue risky projects and increase R&D investment, thereby promoting digital transformation.

First, overconfident managers are affected by the “difficulty effect”. That is, overestimation of their abilities and performance enables managers to believe that they can manage difficult tasks and perform better than the average [37]. Xia, et al. [45] confirmed that overconfidence makes managers willing to adopt more aggressive innovation strategies. Hirshleifer, Low, and Teoh [42] suggested that investing in R&D appeals to overconfident managers because adopting innovative projects may also be viewed as indicative of superior managerial vision.

Second, better-than-average beliefs encourage top managers to pursue a sense of achievement and excellence and thus have a strong enthusiasm and preference for new and high-yield technologies and trends. Such enthusiasm and preference lead to more support for R&D work, and willingness to invest more resources in exploring new technologies and applications. Sharpe, et al. [46] show that overconfident executives are inclined to increase R&D investment, driven by their ambition to pursue innovation and enhance product quality. To sum up, we predict the following:

H2. *Managerial overconfidence is positively associated with organizational R&D investment*.

Digital transformation requires sufficient resources to support it. R&D investment indicates the company can develop or acquire more digital technologies, processes, and products. The improvement of learning and absorption capacity will accelerate digital knowledge absorption and advance digital application, which is more conducive to deepening the level of digital transformation. In addition, Yi, et al. [47] suggest that overconfident managers tend to recruit more passionate and innovative employees. To keep the team’s efforts towards common goals and missions, more attention is paid to the training and education of in-service employees, thereby providing necessary human support for the implementation of digital transformation. Thus, we predict the following:

H3. *R&D investment mediates the relationship between managerial overconfidence and digital transformation*.

### Moderating role of state ownership

State ownership is defined as the percentage of ownership stake that the government holds the majority in a firm [48]. State-owned enterprises (SOEs) are firms with partial (at least 50%) or wholly government ownership [49]. SOEs exist in both developed (e.g., Austria) and emerging markets (e.g., China), and produce approximately half of the world’s annual GDP (World Bank, 2022) [49]. The academic conflict over SOEs and private ownership has long existed. As a result, Chinese SOEs have become a natural experimental battlefield to answer the above debate.

The institutional and the efficiency views are two competing and prevailing perspectives on explaining ownership issues. According to the institutional view, an institutional environment can provide both opportunities and challenges to the operation of a company by generating myriad institutional pressure. Liu, et al. [50] found that Chinese SOEs bear numerous political responsibilities (coercive pressure) to support a national digital transformation strategy that stimulates the digital transformation of companies. Zhou, Gao, and Zhao [48] suggest institutional pressure enables resource allocation. SOEs benefit from inherent political connections with governments that enable companies to gain more resources to invest in R&D activities.

However, the efficiency views argue that compared to private ownership, state ownership is incompatible with efficiency, defined as the degree of transformation of resource input into product output [48]. According to efficiency views, state ownership influences outputs in three important ways. First, the largest shareholder of SOEs is the “state” entity, and government officials at all levels act as agents of the owners, resulting in limited regulatory effects on managerial business decisions compared to POEs [51]. Despite multiple reforms in corporate governance schemes in Chinese SOEs over the past 40 years, executives in some SOEs still lack power constraints [52]. As a result, self-interest behaviors (e.g., corruption) reduce efficiency. Second, the executives of SOEs are appointed by governments more for political reasons. Consequently, SOE top managers are often bureaucrats but not businessmen, lacking the appropriate capabilities or skills to run companies efficiently [48]. Third, over a long period of development, SOEs have taken on the role of improving social welfare, resulting in many redundant resources and outdated routines, which have dragged down organizational efficiency.

Following the efficiency logic, the positive effect of R&D investment and digital transformation may be weak in SOEs. First, although executives in SOEs may increase their investments in digitalization under institutional pressure, they are more likely to engage in self-interested behavior due to agency issues, diverting R&D investment for individual purposes (e.g., official promotion), and reducing the efficiency of converting R&D investment into digital processes and products. Second, firms may suffer from a painful digital transformation period in which investment may not achieve immediate results [26]. When the investment is huge but ultimately fails to achieve actual outcomes, there are accountability risks. Therefore, executives in SOEs are more likely to increase R&D investment in digital projects that can see clear results during their tenure, which reduces the permeation of digital technologies in the organizational process. Third, digital transformation not only requires sufficient resource investment but also new organizational structure and working routines. However, SOEs seek stability in their mindset and existing businesses, which makes them lack motivation and abilities and more likely to fall into dependence on existing resources and work routines. The changes in routines and organizational structure may even trigger internal resistance. Liu, et al. [25] demonstrated that the impact of the R&D intensity of state-owned listed companies on the application of digital technology and the generation of data application scenarios is significantly lower than that of non-state-owned listed companies. To sum up, we predict the following:

H4. *The effect of R&D investment on digital transformation is less positive for state-owned firms than for non-stated firms*.

## Empirical design

### Sample and data

This study uses Chinese A-share listed companies from 2011 to 2021 as the research sample. All data in this article are from the China Stock Market and Accounting Research (CSMAR) database and companies’ annual reports. To improve the reliability of empirical results, we filtered out certain samples as follows. First, we excluded financial companies and the listed companies that were classified as ST, *ST, or PT due to abnormal finance. In the Chinese stock market, ST, * ST, and PT represent companies with continuous financial losses and delisting risks. These types of firms are more likely to reduce their expenses on digital transformation due to profit losses. Therefore, such samples may interfere with the research results. Consistent with Li and Shao [19], this study excluded these samples. Second, following previous studies [32], we winsorized continuous variables at the 1% and 99% quantiles to control for the potential effect of outliers. The final sample consists of 2,884 firms and 20,700 firm-year observations.

### Measurements

#### 1. Independent variables

Following Campbell, et al. [53] and Lee, Park, and Chen [37], we measure managerial overconfidence (Overconfidence) by stock holding behavior. This measure is based on the premise that overconfident top managers tend to hold stocks longer because they have bright views of companies’ future development. Thus, overconfident top managers take a value of 1 if the top management team increases shareholding in the current year (*Overconf1*), and the reason for the increase is not due to stock dividends or rights issues. Otherwise, overconfidence marks 0.

To ensure that our results are robust, we constructed *Overconf2* to measure managerial overconfidence. Previous studies show that overconfident top managers tend to make more acquisitions and investments [54]. Thus, following Malmendier and Tate [54] and Yi, Zhang, and Wang [47], we constructed Eq (1) to estimate the performance of companies’ investment decisions, in which the dependent variable is a growth rate of total assets and the independent variable is a growth rate of operating revenue. We estimated the company residual based on Eq (1) and then subtracted the industry median residual. If the result is greater than 0, represents top managers are overconfident and marked by a value of 1, otherwise is 0.


$$y_{it}=\beta_{0}+\beta_{1}{\left(Sales\;Growth\right)}_{i,t}+\varepsilon_{i,t}$$ (1)

The subscripts *i* and *t* in Eq (1) represent the company and year, respectively. Sales Growth represents the increased rate of operating revenue.

#### 2. Dependent variable

To measure digital transformation (*Digital*), we first utilized Python to download the annual reports of Chinese A-share listed companies from 2011 to 2021. Second, we referred to Zhu, Li, and Ma [23] and constructed a digital dictionary containing 238 keywords. Then, we employed a thesaurus to search and count relevant keywords. Considering that non-negative count data has the characteristic of right skewness, we took a natural logarithm to indicate the level of digital transformation of a company. The larger the value, the higher the degree of digital transformation of the enterprise.

#### 3. Mediating and moderating variables

We use the R&D expenses as a proxy of R&D investment (*RD*). To mitigate the gap in R&D investment between enterprises and industries, *RD* was operationalized as the logarithm of R&D expenses.

Following Zhou, Gao, and Zhao [48], we created a dummy variable to measure state ownership (*SOE*). If a firm is majorly owned by the local governments and central government, mark 1, otherwise 0.

#### 4. Control variables

To control for possible confounding factors that may influence the level of digital transformation, we controlled the following variables. The measurement of controls is shown in Table 1.

**Table 1:** Measurement of the variables.

Variables	Symbol	Measurement
Digital transformation	*Digital*	Ln (the total frequency of relevant keywords + 1)
Managerial overconfidence	*Overconf1*	Mark 1 if the number of management’s shares increased in that year, and the reason for the increase was not a stock dividend or rights issue; otherwise 0.
	*Overconf2*	Mark 1 if the value of the estimated company’s residuals minus the industry median is greater than 0; otherwise 0.
R&D investment	*RD*	Ln (R&D expenses + 1)
State ownership	*SOE*	Mark 1 if the firm is state-owned; otherwise 0
Shareholding ratio of the largest shareholder	*Top1*	Shareholding number of the largest shareholder/total shares
Firm age	*Age*	Ln (time of the year—time of listing date+1)
Capital intensity	*Capins*	Total assets/operating revenue
Asset-liability ratio	*Lev*	Liabilities/total assets
Profitability	*ROA*	Net profit/total assets
Chair-CEO duality	*Dual*	Mark 1 if the chairman is also the general manager; otherwise, 0
Board size	*Board*	Ln (Number of directors)
Independent directors ratio	*Ind*	Number of independent directors/number of directors
Cash	*Cash*	Cash Holdings/total assets
Individual factors	*Individual*	Control for individual company
Year factor	*Year*	Control for year factor

### Model setting and empirical strategies

To examine hypothesis 1, we constructed Eq (2) to estimate the impact of managerial overconfidence on digital transformation. Considering the possibility of time delay in decision-making effectiveness, we employed a one-year lag for the independent variables in the models. Where *Digital_i,t_* represents the level of digital transformation of *i* company in *t* year, *Overconfidence*_*i,t*−1_ represents the managerial overconfidence of *i* company in *t-1* year, ∑*Controls_i,t_* represents the control variables at the enterprise level. ∑*Firm* and ∑*Year* control for individual firm and year effects and *ε_i,t_* is the error term. If α_1_ is positive and significant, H1 is confirmed.


$${Digital}_{i,t}=\alpha_{0}+\alpha_{1}{Overconfidence}_{i,t-1}+\sum {Controls}_{i,t}+\sum Firm+\sum Year+\varepsilon_{i,t}$$ (2)

To examine H2 and H3, we constructed the Eqs (3) and (4). If the following conditions are simultaneously satisfied, then R&D investment (*RD*) plays a mediating role between managerial overconfidence and digital transformation: β_1_ in Eq (3) is significant, γ_1_ and γ_2_ in Eq (4) are significant, and γ_1_ in Eq (4) is lower than α_1_ in Eq (2).


$${RD}_{i,t}=\beta_{0}+\beta_{1}{Overconfidence}_{i,t-1}+\sum {Controls}_{i,t}+\sum Firm+\sum Year+\varepsilon_{i,t}$$ (3)

$${Digital}_{i,t}=\gamma_{0}+\gamma_{1}{Overconfidence}_{i,t-1}+{\gamma_{2}RD}_{i,t}+\sum {Controls}_{i,t}+\sum Firm+\sum Year+\varepsilon_{i,t}$$ (4)

Finally, to test the moderating effect of state ownership, the hierarchical test method is used to perform the empirical analysis, as shown in Eq (5). If γ_4_ is positive and significant, H4 is confirmed.


$${Digital}_{i,t}=\gamma_{0}+\gamma_{1}{Overconfidence}_{i,t-1}+{\gamma_{2}RD}_{i,t}+\gamma_{3}{SOE}_{i,t}+\gamma_{4}{SOE}_{i,t}\times {RD}_{i,t}+\sum {Controls}_{i,t}+\sum Firm+\sum Year+\varepsilon_{i,t}$$ (5)

## Analysis and results

### Descriptive statistics

Table 2 presents the descriptive statistics of the variables. The mean value of digital transformation is 1.52, the standard deviation is 1.445, and the median is less than the mean, indicating the level of digital transformation with certain right skewness, that is, some companies have a high degree of digital transformation. Additionally, the minimum value is 0, and the maximum value is 5.147, indicating a significant difference in digitalization and some companies have not yet undergone digital transformation. The mean values of *Overconf1* and *Overconf2* are 0.180 and 0.500, and the standard deviations are 0.384 and 0.500, respectively, indicating that executive over-confidence varies among different enterprises. The mean and median values of the other variables are relatively close, indicating that the variables are approximately symmetrically distributed.

**Table 2:** Descriptive statistical analysis of variables (N = 20700).

Variables	Mean	S.D.	Min	Median	Max
*Digital*	1.520	1.445	0	1.386	5.147
*Overconf1*	0.180	0.384	0	0	1
*Overconf2*	0.500	0.500	0	0	1
*Top1*	0.332	0.144	0.087	0.310	0.751
*Age*	2.257	0.685	0.693	2.303	3.367
*Capins*	2.534	2.171	0.401	1.935	15.890
*Lev*	0.423	0.198	0.051	0.417	0.895
*ROA*	0.035	0.063	-0.250	0.035	0.204
*Dual*	0.282	0.450	0	0	1
*Board*	2.287	0.250	1.609	2.303	2.890
*Ind*	0.385	0.074	0.250	0.375	0.600
*Cash*	0.189	0.130	0.017	0.155	0.734

### Regression model

A unit root is a stochastic trend in a time series, which can cause spurious regressions. Thus, we first performed the Fisher unit root test. To carry out this test, the level of significance of the ADF statistics for cross-section unit i is used. This type of test is attractive because it is robust and can be used in an unbalanced panel. The statistics results of dependent variable, independent variable, mediator, and moderator reject null hypotheses (p<0.001), which means that the data do not contain unit roots. Therefore, we can further run regression models.

Then, we performed the Hausman test. The test result is significant, rejecting the null hypothesis. Therefore, it is reasonable to use a fixed effects model for regression.

The estimated results of the benchmark regression model are shown in Table 3. As shown in columns (1) and (2), the coefficients of *Overconf1* and *Overconf2* on digital transformation are 0.031 and 0.022 (p<0.05), respectively. Columns (3) and (4) are estimated by adding control variables, as shown that the coefficients of *Overconf1* and *Overconf2* on digital transformation are 0.032 and 0.023, respectively. Both are significant at the 5% level. These findings support H1, that is, managerial overconfidence has a positive and significant impact on digital transformation.

**Table 3:** The benchmark regression result.

Variables	(1) Digital	(2) Digital	(3) Digital	(4) Digital
*Overconf1*	0.031**		0.032**	
	(0.014)		(0.014)	
*Overconf2*		0.022**		0.023**
		(0.010)		(0.010)
*Top1*			-0.289***	-0.288**
			(0.112)	(0.112)
*Age*			0.206***	0.210***
			(0.037)	(0.037)
*Capins*			-0.013***	-0.013***
			(0.005)	(0.005)
*Lev*			0.172**	0.161**
			(0.067)	(0.067)
*ROA*			0.206*	0.192
			(0.117)	(0.117)
*Dual*			0.028	0.027
			(0.019)	(0.019)
*Board*			0.163***	0.163***
			(0.029)	(0.029)
*Ind*			-0.212**	-0.210**
			(0.088)	(0.088)
*Cash*			-0.056	-0.056
			(0.069)	(0.069)
*Individual*	yes	yes	yes	yes
*Year*	yes	yes	yes	yes
*R* ^*2*^	0.818	0.818	0.820	0.820
*N*	20513	20513	20513	20513

*** p < 0.01, ** p < 0.05, * p < 0.1. Unstandardized coefficients are reported. Standard errors are in parentheses. The same is below.

### Mediating effect of R&D investment

To test H2 and H3, that is, whether managerial overconfidence improves the degree of digital transformation through R&D investment. As shown in columns (1) and (3) of Table 4, the influence coefficients of *Overconf1* and *Overconf2* on *RD* are 0.041 and 0.071 (p<0.01), respectively. It shows that managerial overconfidence can promote the increase of R&D investment, and the H2 is verified. Columns (2) and (4) examine the combined impact of managerial overconfidence and R&D investment on digital transformation. As shown in columns (2) and (4) of Table 4, the coefficients of *Overconf1* and *Overconf2* on digital transformation are 0.029 and 0.023 (p<0.05), respectively. We calculated the mediation effect and found that the proportion of the mediating effect in the total effect is 0.15 and 0.35, respectively, indicating that managerial overconfidence can indirectly affect digital transformation by increasing R&D investment. Therefore, H3 is supported.

**Table 4:** Mediation effect test.

Variables	(1) RD	(2) Digital	(3) RD	(4) Digital
*Overconf1*	0.041***	0.029**		
	(0.012)	(0.014)		
*Overconf2*			0.071***	0.023**
			(0.009)	(0.011)
*RD*		0.117***		0.116***
		(0.011)		(0.011)
*Top1*	-0.073	-0.335***	-0.069	-0.334***
	(0.137)	(0.124)	(0.137)	(0.124)
*Age*	0.016	0.148***	0.026	0.151***
	(0.036)	(0.040)	(0.036)	(0.040)
*Capins*	-0.070***	0.003	-0.072***	0.003
	(0.007)	(0.006)	(0.007)	(0.006)
*Lev*	0.500***	0.107	0.468***	0.097
	(0.073)	(0.074)	(0.073)	(0.075)
*ROA*	1.078***	0.143	1.032***	0.130
	(0.119)	(0.122)	(0.119)	(0.122)
*Dual*	0.030*	0.016	0.028*	0.015
	(0.017)	(0.021)	(0.017)	(0.021)
*Board*	0.147***	0.128***	0.148***	0.129***
	(0.027)	(0.031)	(0.026)	(0.031)
*Ind*	-0.215***	-0.202**	-0.216***	-0.202**
	(0.082)	(0.093)	(0.082)	(0.093)
*Cash*	-0.060	-0.026	-0.060	-0.026
	(0.059)	(0.072)	(0.059)	(0.072)
*Individual*	yes	yes	yes	yes
*Year*	yes	yes	yes	yes
*R2*	0.881	0.834	0.882	0.834

### Moderating effect of state ownership

To examine H4, we first standardized the variables to reduce the multi-collinearity problem and then calculated the interaction term. Column (1) and column (2) in Table 5 show that the coefficients of the interaction term between RD and SOE are negative and significant (β_1_ = -0.074, p < 0.01; β_2_ = -0.073, p < 0.01), while the mediating effect is still significant, indicating that state ownership weakens the second half of mediation effect. That is, the effect of R&D investment on digital transformation is less positive for state-owned firms than for non-stated firms. Therefore, H4 is supported.

**Table 5:** Moderating effect test.

Variables	(1) Digital	(2) Digital
*Overconf1*	0.022	
	(0.015)	
*Overconf2*		0.017
		(0.011)
*SOE*	1.189***	1.187***
	(0.337)	(0.337)
*RD*	0.144***	0.144***
	(0.015)	(0.015)
*RD×SOE*	-0.074***	-0.073***
	(0.019)	(0.019)
*Top1*	-0.316**	-0.315**
	(0.131)	(0.130)
*Age*	0.125***	0.127***
	(0.043)	(0.043)
*Capins*	0.006	0.006
	(0.007)	(0.007)
*Lev*	0.075	0.067
	(0.078)	(0.078)
*ROA*	0.111	0.101
	(0.127)	(0.127)
*Dual*	0.006	0.005
	(0.021)	(0.021)
*Board*	0.124***	0.125***
	(0.032)	(0.032)
*Ind*	-0.201**	-0.201**
	(0.096)	(0.096)
*Cash*	-0.046	-0.045
	(0.075)	(0.075)
*Individual*	yes	yes
*Year*	yes	yes
*R* ^*2*^	0.836	0.836
*N*	16862	16862

### Robustness test

We took two steps to address the cross-sectional dependence concern. First, considering that panel data may have a problem of residual cross-sectional dependence, we estimated fixed-effect models with a weak cross-sectional dependence test (i.e., Friedman’s test) on the regression errors. The statistics results of all models show that the p-value is greater than 0.1, suggesting that no significant cross-sectional dependence problem. Second, previous studies suggest that the Driscoll-Kraay estimator is robust to general forms of spatial and temporal correlation (e.g., [55]). Thus we estimate Driscoll-Kraay standard errors and the outcomes are consistent with the results in Tables 3 and 4. Therefore, our results are still solid.

We also addressed endogeneity problems. First, our study used the fixed effects model to alleviate endogeneity to some extent. The reason for using a fixed effects model is due to the presence of individual effects, time effects, and explanatory variables. If a fixed effects model is not used, the individual and temporal influences will slip into the perturbation term, leading to endogeneity issues. Second, to mitigate endogeneity issues caused by reverse causality, we further considered the lag effect of overconfidence and lag 2 years and obtained highly consistent results. The results are in line with predictions. Third, we used an instrumental variable approach to mitigate endogeneity caused by missing important variables. On the basis of theoretical antecedents of the overconfidence of top managers, we chose the mean overconfidence value of other companies in the same industry within the same year (Mean_ind). In line with previous studies (i.e., [56]), we argue that companies in the same industry may face similar industry and external environments, and thus it is positively correlated with overconfidence. As shown in Table 6, the instrument strongly and positively affects the overconfidence of top managers. Cragg-Donald Wald F statistic is greater than Stock-Yogo weak ID test critical values, which means that the instrument is not weakly identified. In the second stage of estimation, the overconfidence of top managers significantly and positively affects digital transformation. Overall, the finding in this study is robust.

**Table 6:** Endogeneity test: Instrumental variable approach.

Variables	(1) Overconf1	(2) Digital
*Mean_ind*	0.539***	
	(0.026)	
*Overconfi1*		0.767***
		(0.097)
*Top1*	0.002	-0.159
	(0.052)	(0.105)
*Age*	0.004	1.076***
	(0.010)	(0.019)
*Capins*	0.003	-0.001
	(0.002)	(0.005)
*Lev*	-0.012	-0.101
	(0.033)	(0.065)
*ROA*	0.044	0.161
	(0.060)	(0.120)
*Dual*	-0.011	0.045**
	(0.010)	(0.020)
*Board*	-0.007	0.189***
	(0.015)	(0.306)
*Ind*	0.018	-0.219**
	(0.047)	(0.093)
*Cash*	-0.021	0.008
	(0.033)	(0.067)
*Individual*	Yes	Yes
*Angrist-Pischke multivariate F test*	423.56***	-
*Anderson canon*. *corr*. *LM statistic*	413.83***	413.83
*Cragg-Donald Wald F statistic*	423.56	423.56
*N*	20468	20468

## Conclusions

Drawing from the upper-echelon theory and digital transformation literature, we propose that overconfidence as a cognitive bias of the top management team will affect corporate digital transformation. Specifically, we argue that overconfident top managers overly assess their ability and performance under uncertainty and will be inclined to bear more challenging tasks than the average thereby investing more in R&D activities to advance digital transformation. We also argue that state ownership will weaken the positive relationship between R&D investment and digital transformation. All hypotheses are confirmed by a panel data set collected from Chinese-listed companies from 2011–2021. To be specific, empirical tests prove that managerial overconfidence in top management teams exhibits a positive relationship with corporate digital transformation and the relationship is mediated by R&D investment. In addition, we find that compared to state-owned companies, R&D investment has a more significant positive effect on digital transformation in non-state-owned companies.

### Theoretical implications

With this comprehensive examination, we contribute to the existing literature in three distinct ways. First, our study contributes to the digital transformation literature by being one of the first to incorporate the overconfidence of decision-makers into digital transformation. As previous studies suggest, digital transformation involves risky and discontinuous changes in organizational structure, business processes, and micro routines, which need the positive role of top managers in systems implementation [23]. Building on this reality, we highlight the bounded rationality of the top management team and take overconfidence, the “mother of all biases”, into account. In so doing, we address the fact that the overly assessment of their ability, knowledge, and performance can inspire top managers to make bold decisions to invest in digitalization and then steer through the uncertain period when digital transformation may not produce immediate benefits. Therefore, our findings confirm the critical role of cognitive bias in explaining corporate strategic decision-making and changes but also go beyond the studies that only highlight the individual top manager [23].

Second, by introducing R&D investment as a mediator of digital transformation, we attempt to identify the pathways for advancing digital transformation more clearly. Prior digital transformation studies show that companies tend to increase R&D activities to provide more technical support for the company’s digital transformation [32, 57], however, they rarely explain the factors driving R&D activities. Our theoretical framework assumes and verifies that overconfidence leads to biased decision-making, resulting in a higher likelihood of risk-taking and innovation input. We integrate overconfidence and digital transformation literature and suggest that the overconfidence of top managers explains companies’ decision to increase R&D investment, which finally facilitates corporate digital transformation. Therefore, we identify and expound the pathway for advancing digital transformation from the perspective of R&D investment.

Third, striving to provide a more comprehensive understanding of the relationship between managerial overconfidence and digital transformation, the moderating effect of state ownership is explored. Our findings confirm the boundary condition of state ownership in the relationship between R&D investment and digital transformation. Our findings prove the efficiency paradox in Chinese state-owned companies and also inform the research that Chinese state-owned companies suffer from underperformance in transforming R&D investment into substantial output that firms need in the digitalization era.

### Managerial implications

Our findings offer some important implications for managers and policymakers. First, managers should attach importance to the construction of executive teams and fully recognize the impact of executive characteristics on the development of the companies. Overconfident executives are seen as hubris and risk tolerance, while they have a strong impact on their followers in the implementation process. The trait of overconfidence is beneficial for executives to overcome various difficulties and obstacles in organizational change. When optimizing the configuration of the executive team, companies should pay attention to the complementarity of the background, professional knowledge, and abilities of executives, and build a moderately overconfident team.

Second, the impact of overconfident executives on corporate digital transformation depends on specific value-creation actions, with R&D investment being a crucial factor. Overconfidence may encourage companies to embrace open innovation with partner companies to solve digital resource constraints, however, R&D investment is necessary. With the increase in R&D investment, companies can introduce more advanced technological products and processes, and improve the level of digital transformation.

Third, digital transformation is a key driving force for the long-term growth of state-owned enterprises. We found that compared to state-owned companies, R&D investment has a more significant positive effect on digital transformation in non-state-owned companies. This may be explained by efficiency problems. As a result, mix-ownership reform may be the one of solutions. The introduction of non-state shareholders via mixed-ownership reform will reduce the policy-based burden of companies and then increase the exposure to market competition, which encourages the companies to increase efficacy [58].

### Limitations and further research

This study has several caveats that should offer opportunities for future research. First, because executive overconfidence is a periodic personal characteristic influenced by self-attribution and power structure, future research can further examine the dynamic evolution of executive overconfidence from a more micro perspective and its role in the digital transformation process.

Second, according to upper-echelon theory, the strategic decision-making of managers is influenced by the internal and external environment of the organization. Therefore, more moderating factors can be added to explore the relationship between managerial overconfidence and digital transformation in the future, such as environmental dynamism and internal resource redundancy.

Third, this study only uses the frequency of characteristic words disclosed in annual reports to measure the level of digital transformation, which may differ from the actual transformation situation of companies. Therefore, in the future, a combination of qualitative and quantitative methods may be considered to comprehensively evaluate the level of digital transformation.
